# Supplementary material for: Biofabricating murine and human myo‐substitutes for rapid volumetric muscle loss restoration
Source: EMBO Mol Med. 2021 Feb 15;13(3):e12778. doi: 10.15252/emmm.202012778 (PMC7933978; doi:10.15252/emmm.202012778)
Supplement: Supplementary file 1 — Appendix [file EMMM-13-e12778-s001.pdf]

Appendix

Appendix Figure S1. Acellular 3D-printed construct characterization upon implantation at different time points.

Appendix Figure S2. Fluorescence image and relative ImageJ output analysis employed for vessel density evaluation.

Appendix Figure S3. Vascularization assessment on TA reconstructed upon 3D wet-spun myo- substitute implantation.

Appendix Figure S4. Electrophysiological set-up.

Appendix Table S1. Absolute force raw data obtained from electrophysiological set-up.

Appendix Table S2. Raw data of macrophage positive area per ROI (40000 μm^2), for the three experimental conditions.

Appendix Table S3. Raw data of pre- and post-synaptic elements manually counted per ROI (125000 μm^2).

Appendix Table S4. Raw data of NADH-TR assay counting the relative number of oxidative fibers respect the total number of fibers.

Appendix Table S5. Raw data of SMA and vW positive area per ROI (1 mm^2), for native and grafted TA.

Appendix Table S6. Raw data of the number of vessels manually counted per ROI (0,2 mm^2).

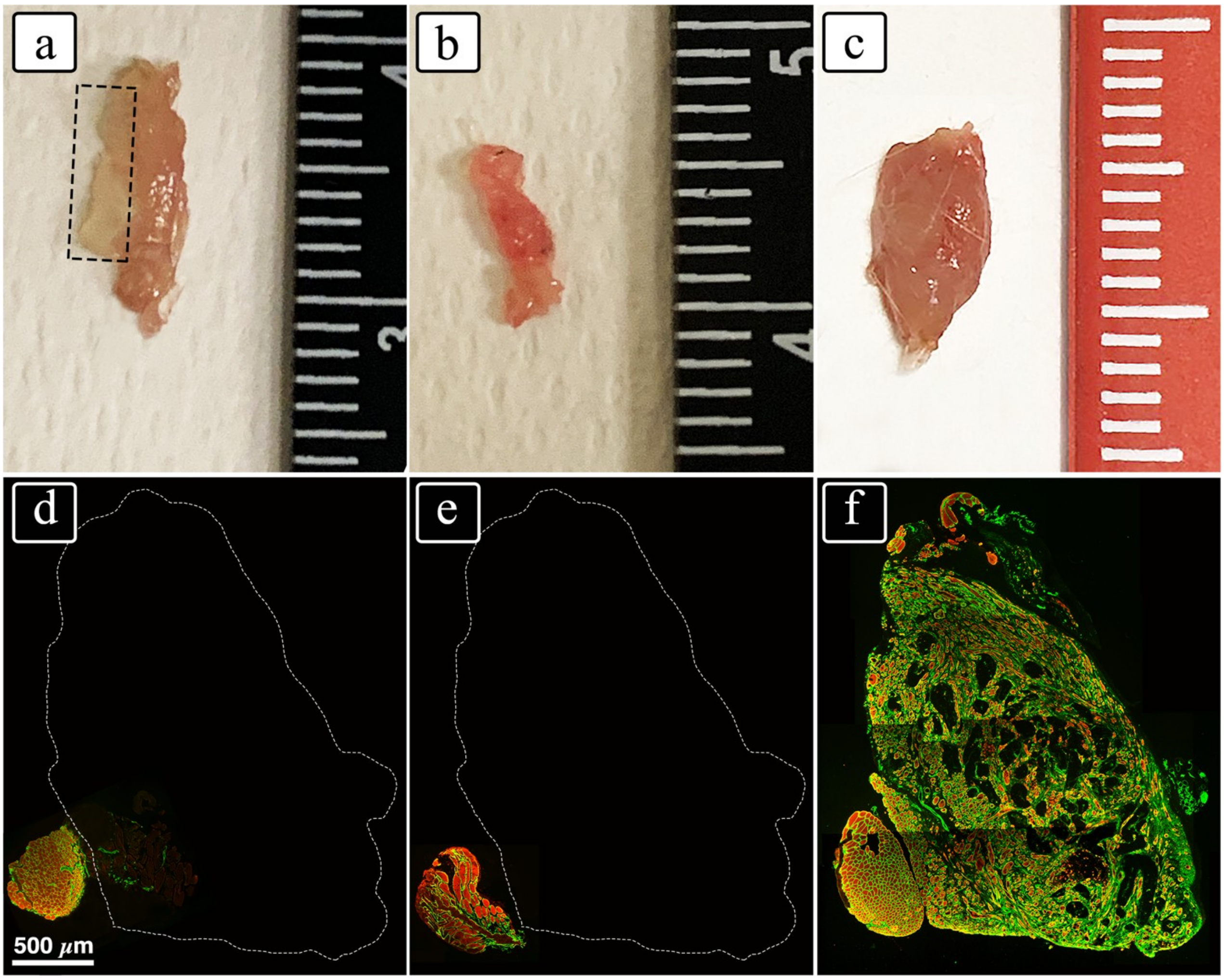

Appendix Figure S1. Acellular bioink has been 3D-printed and characterized upon implantation. a-c) Macroscopic comparison after TA isolation performed after 10 (a) and 20 days (b) from acellular construct implantation compared to Mabs-derived reconstructed TA 20 days upon graft (c). d -f) Immunofluorescence analysis against MHC (red) and Laminin (green) on TA sections from acellular derived construct implantation at 10 (d) and 20 days (e) and Mabs derived myo-substitute implantation at 20 days upon graft (f).

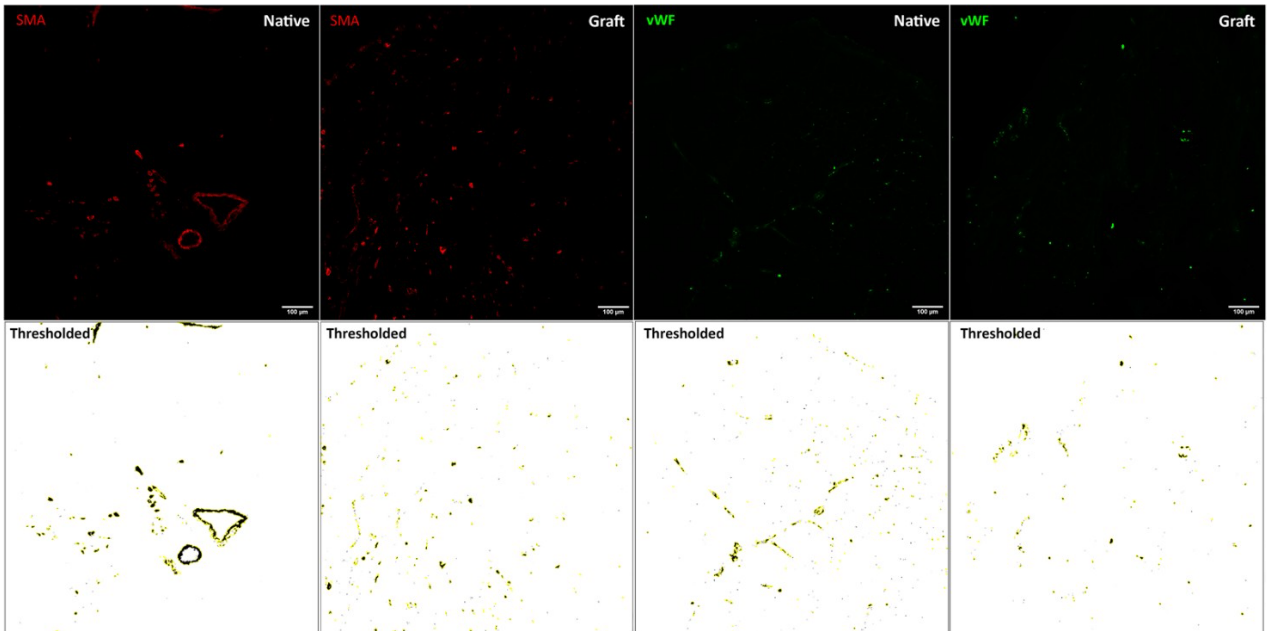

Appendix Figure S2. Fluorescence image and relative ImageJ output analysis employed for vessel density evaluation. Cross sections from normal muscle tissue (native) and engrafted reconstructed tissue (graft) were immunoassayed for SMA (red) or vW (green), both vessel markers. Corresponding thresholded images (below) in black and white show in yellow the selection outlines generated with ImageJ (*Image>Adjust>Threshold*, then *Analyze>Analyze particles>Outlines*) to calculate the signal global area.

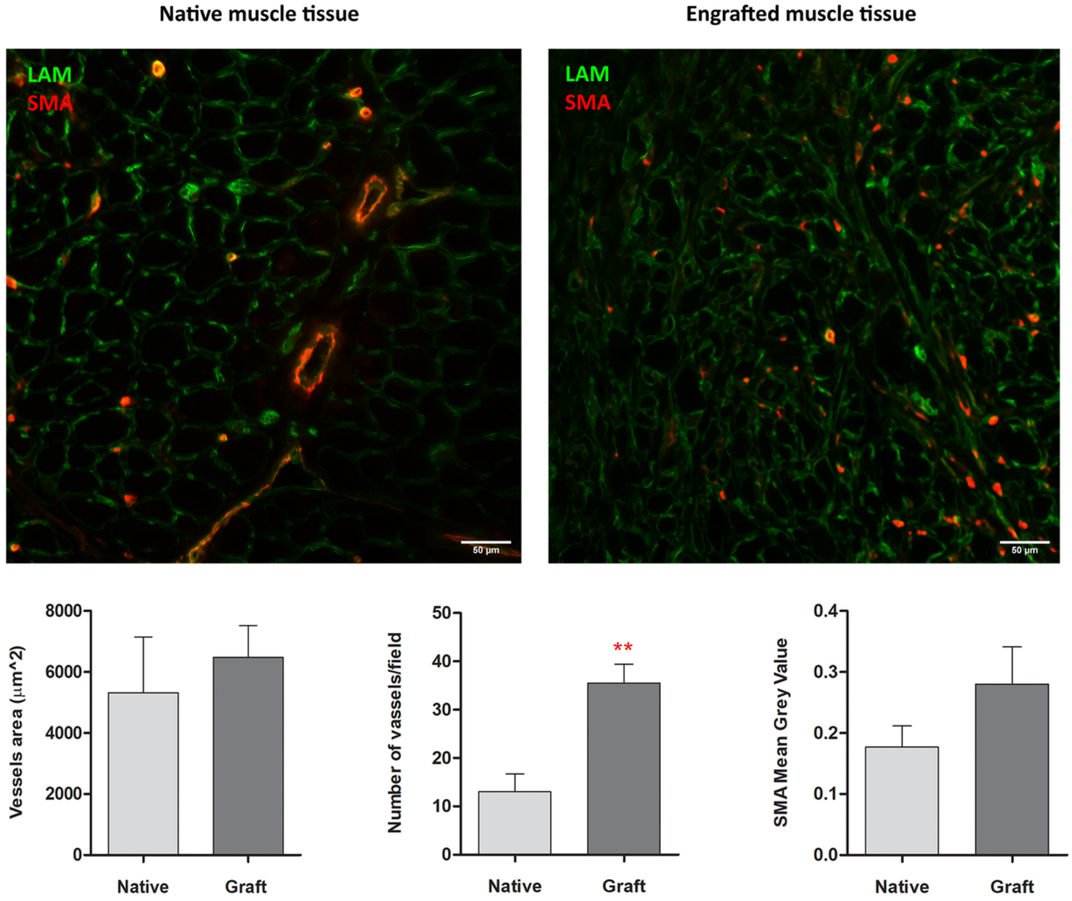

Appendix Figure S3. Vascularization assessment on TA reconstructed upon 3D wet-spun myo-substitute implantation. Immunofluorescence on cross sections from normal TA muscle (native) and engrafted reconstructed tissue (graft) against SMA (red) and Laminin (green) for vessel density evaluation by means of Vessel area, number of vessel and SMA fluorescence intensity is reported in the diagram as means ± S.D. and statistical significance was analyzed by Anova test (P < 0.05 was considered significant: *=0,0019).

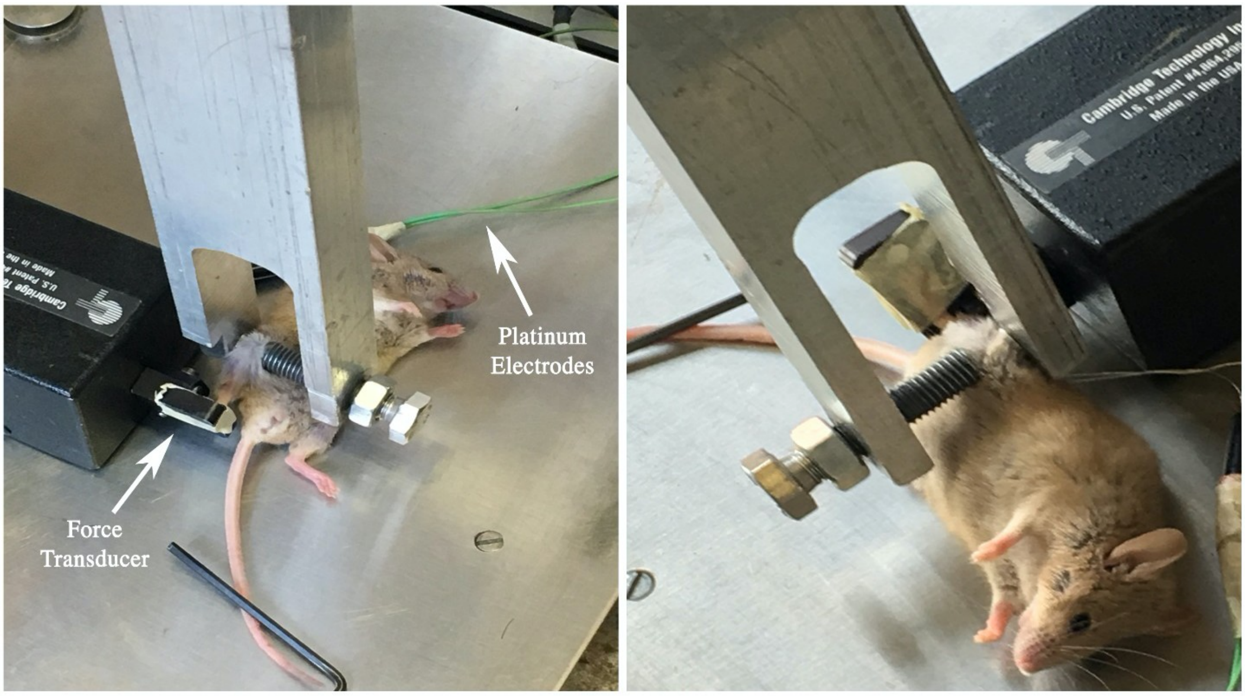

Appendix Figure S4. Electrophysiological set-up used to assess force recovery.

	Freq. (Hz)	Force (N)						
		**1**	**2**	**3**		**average**	**s.d.**	**s.e.**
**Graft (Wet spun)**	**4**	0,37	0,51	0,92		**0,6**	**0,28**	**0,14**
	**20**	0,49	0,61	1,10		**0,73**	**0,32**	**0,16**
	**40**	0,98	1,21	1,89		**1,36**	**0,47**	**0,24**
	**55**	1,44	1,78	2,11		**1,78**	**0,33**	**0,17**
	**75**	1,88	2,22	2,45		**2,18**	**0,28**	**0,14**
	**100**	2,21	2,45	2,89		**2,52**	**0,35**	**0,17**
	**150**	2,15	2,41	2,75		**2,44**	**0,30**	**0,15**
		**1**	**2**	**3**	**4**	**average**	**s.d.**	**s.e.**
**Control**	**4**	0,36	0,61	0,71	0,32	**0,5**	**0,19**	**0,10**
	**20**	0,42	0,88	0,75	0,39	**0,61**	**0,24**	**0,12**
	**40**	0,83	1,22	1,09	0,75	**0,97**	**0,22**	**0,11**
	**55**	0,98	1,48	1,35	0,99	**1,20**	**0,25**	**0,13**
	**75**	1,01	1,58	1,51	1,08	**1,29**	**0,29**	**0,15**
	**100**	1,12	1,70	1,65	1,11	**1,39**	**0,32**	**0, 16**
	**150**	1,10	1,68	1,68	1,10	**1,39**	**0,34**	**0,17**
		**1**	**2**	**3**	**4**	**average**	**s.d.**	**s.e.**
**Wild Type**	**4**	0,73	0,73	0,42	0,57	**0,62**	**0,13**	**0,06**
	**20**	0,79	0,73	0,45	0,58	**0,64**	**0,13**	**0,06**
	**40**	0,75	0,78	0,67	0,73	**0,73**	**0,05**	**0,02**
	**55**	1,67	1,32	1,18	1,37	**1,20**	**0,16**	**0,07**
	**75**	2,53	2,75	2,07	2,78	**2,47**	**0,32**	**0,14**
	**100**	3,03	3,37	3,08	3,38	**3,21**	**0,16**	**0,07**
	**150**	3,23	3,67	3,28	3,67	**3,47**	**0,21**	**0,09**

Appendix Table S1. Absolute force raw data obtained from electrophysiological set-up.

% of macrophage positive area

wt	10 days	20 days
0,193	6,873	7,639
0,283	5,436	4,410
0,423	5,852	3,639
0,302	4,738	2,079
0,440	3,051	3,194
0,443	1,853	1,872
0,391	4,420	1,364
0,489	6,187	1,428
0,476	11,257	2,851
0,512	6,440	2,384

Appendix Table S2. Raw data of macrophage positive area per ROI (40000 μm^2), for the three experimental conditions.

% of coupled synaptic elements

	Syn	BTX	coupled
	4	2	2
	3	3	3
	6	6	6
	3	2	2
	10	8	8
	6	8	6
	7	3	3
Total	**39**	**32**	**30**
% of coupled	**77**	**94**	

Appendix Table S3. Raw data of pre- and post-synaptic elements manually counted per ROI (125000 μm^2).

% of oxidative fibers

wt	20 days
60,19	52,48
53,19	35,71
63,34	39,33
37,42	23,52
37,58	28,81

Appendix Table S4. Raw data of NADH-TR assay counting the relative number of oxidative fibers respect the total number of fibers.

Marker-positive area (*μ*m^2)

native		graft	
SMA	vW	SMA	vW
4207,14	4314,32	5575,09	2294,44
8897,10	3339,65	5296,60	2683,55
2857,07	3011,12	8562,10	2851,12

Appendix Table S5. Raw data of SMA and vW positive area per ROI (1 mm^2), for native and grafted TA.

Number of vassels/0,2 mm^2

Native		graft	
SMA	vW	SMA	vW
10	41	46	45
12	52	41	28
9	47	34	43

Appendix Table S6. Raw data of the number of vessels manually counted per ROI (0,2 mm^2).
