# Supplementary material for: Biofabricating murine and human myo‐substitutes for rapid volumetric muscle loss restoration
Source: EMBO Mol Med. 2021 Feb 15;13(3):e12778. doi: 10.15252/emmm.202012778 (PMC7933978; doi:10.15252/emmm.202012778)
Supplement: Supplementary file 2 — Expanded View Figures PDF [file EMMM-13-e12778-s002.pdf]

Expanded View Figures

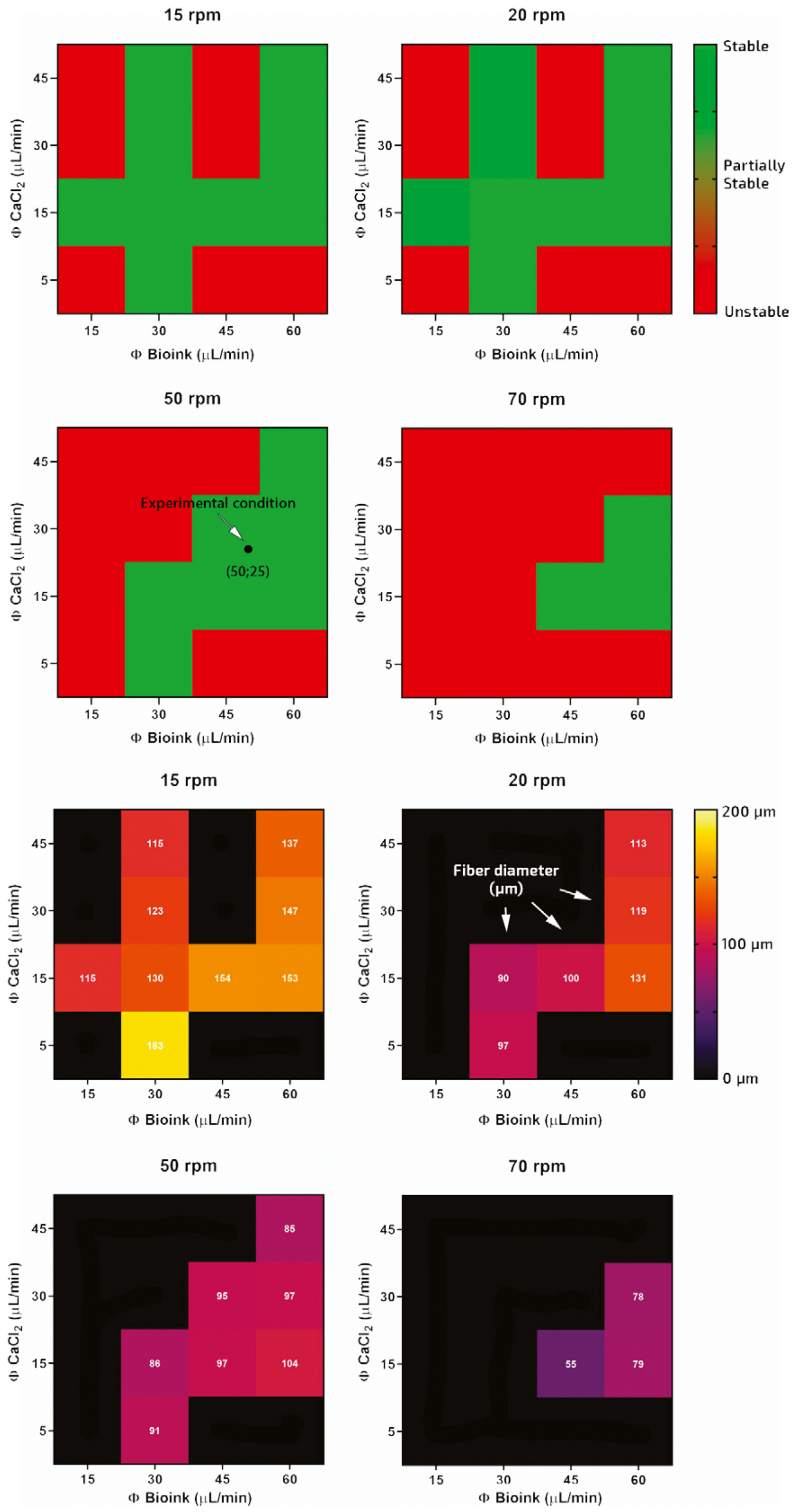

Figure EV1. Characterization of the wet-spinning bioprinting system.

The upper panels show the stability of the system for different flow rates of calcium chloride (5–45 µl/min) and bioink (15–60 µl/min) and for different spinning speed (15–70 rpm). The lower panels show the relative size of the spun fibers (in white displayed within each square).

© 2021 The Authors

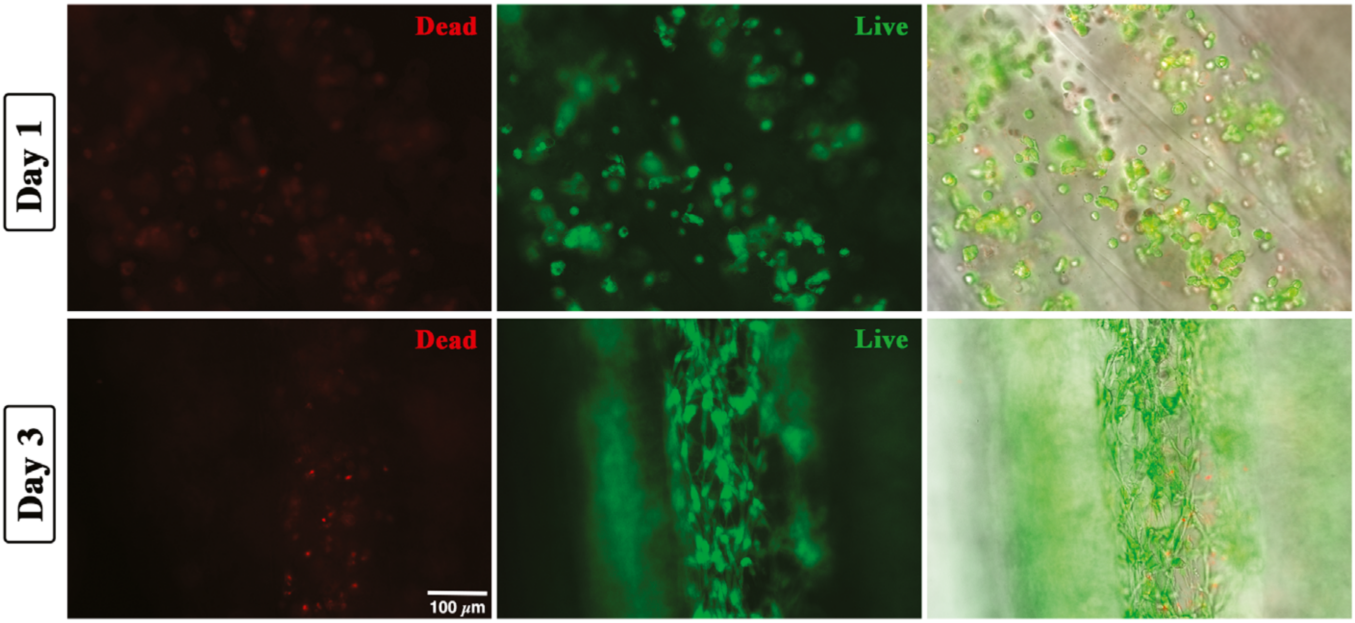

Figure EV2. Live/dead staining of yarns loaded with mouse primary cells (Mabs).

Fluorescence images showing labeled Mabs with calcein-AM (viable cells, green) and propidium iodide (dead cells, red), respectively, on the left and middle of the panel. Superimposition of fluorescent signal over phase-contrast photograph on the right. For the sake of clarity, samples used for live/dead investigation were prepared out of few layers of fibers to favor fluorescence cell imaging.

© 2021 The Authors

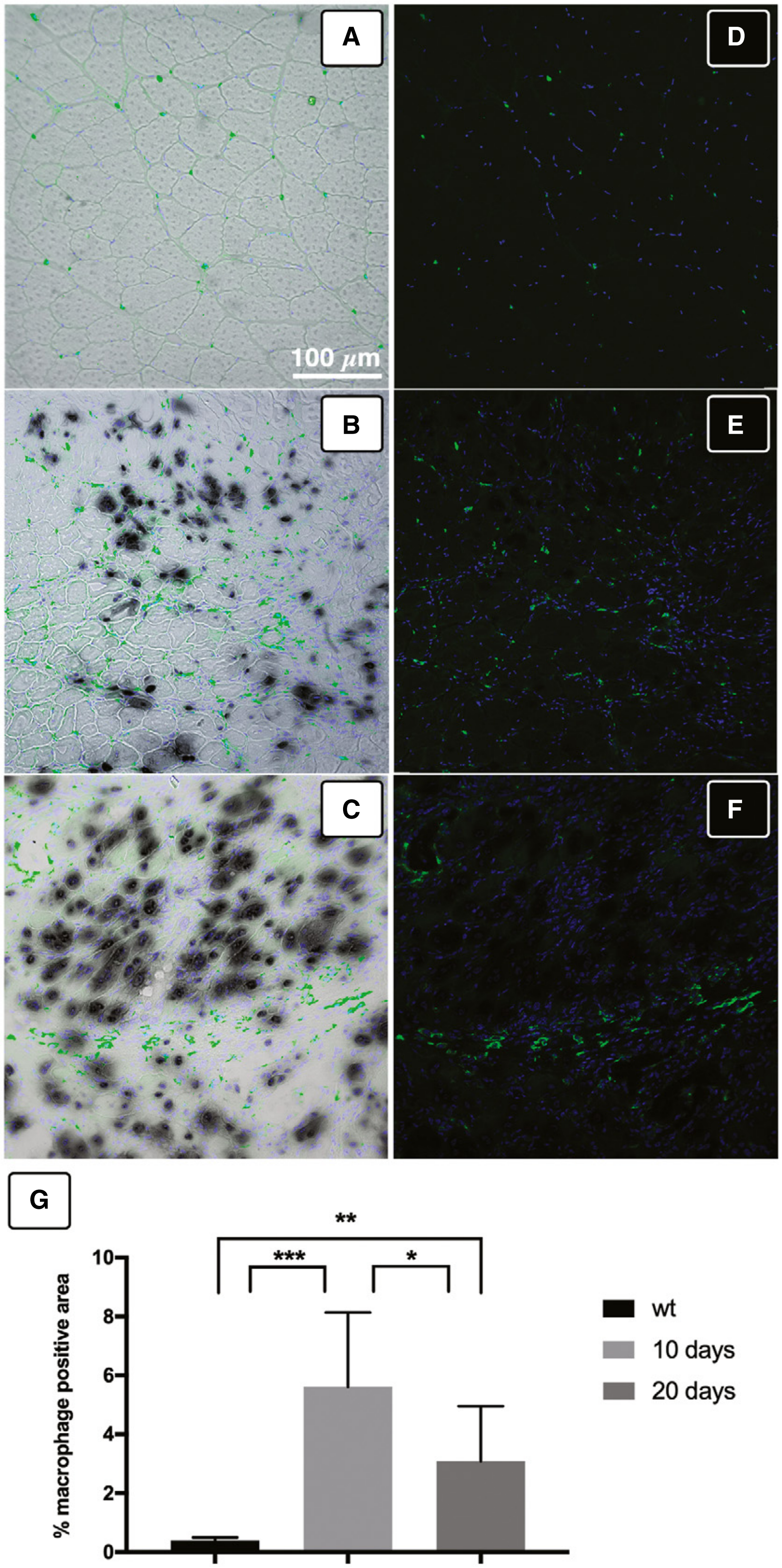

Figure EV3. Immunofluorescence analysis of macrophage infiltration upon myo-substitute implantation.

A, D Wild-type TA.
B, E 10 days after implantation.
C, F 20 days upon graft. Antibody against mannose receptor (green) has been used to label macrophages (D and E), and the staining has been superimposed on phase-contrast image upon X-Gal staining (A–C). Nuclei were counterstained with DAPI (blue).
G Macrophage infiltration comparison obtained evaluating wt and implanted section at different time points: Diagram representing the rate of positive mannose receptor area is expressed as means ± SD, and statistical significance was analyzed by ANOVA test ($P < 0.05$ was considered significant: WT vs. 10 days *** = 0.0001; WT vs. 20 days ** = 0.0072; 10 days vs. 20 days * = 0.0119).

 © 2021 The Authors

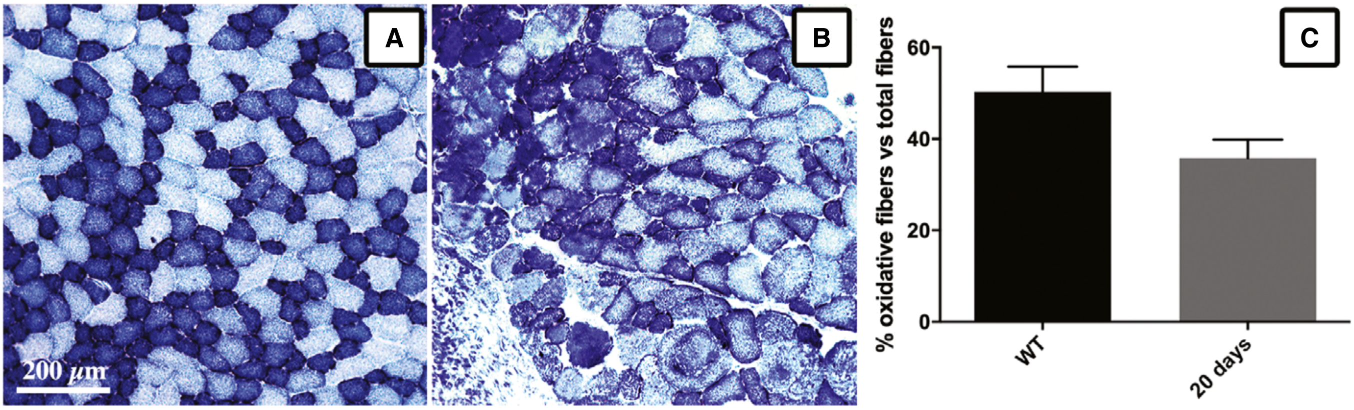

Figure EV4. NADH reductase assay on wt and myo-substitute reconstructed mouse TA.

A, B wt TA (A) and reconstructed TA upon myo-substitute engraftment (20 days) (B) have been stained for NADH-TR labeling type I slow oxidative fibers (dark blue) and type II fast glycolytic fibers (light blue).

C Graph indicating the rate of oxidative (dark blue) fibers scored in different section fields, representing the means ± SD; any significance has been revealed upon Student's *t*-test analysis.

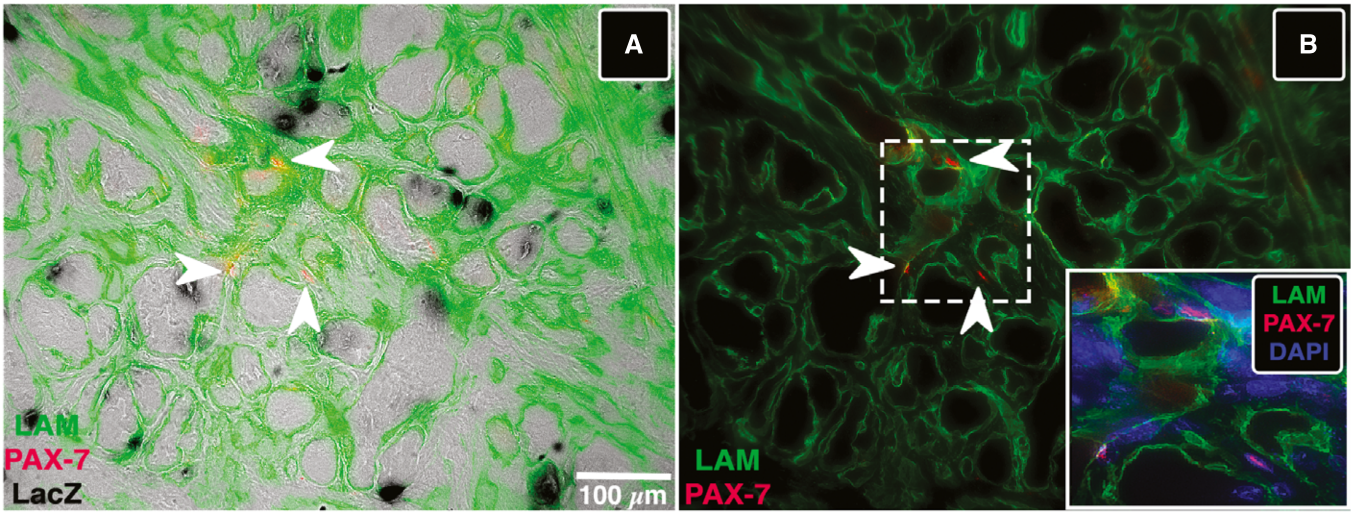

Figure EV5. Satellite cells Pax-7 labeling on reconstructed TA sections.

A Immunofluorescence image resulting by LAM (green) and PAX-7 (red) antibody reactions superimposed to LacZ staining showing Pax-7-positive satellite cells (arrowheads) in the Mab-derived artificial TA.

B Fluorescence image from (A) inset from dashed area shows Pax-7 signal (arrowheads) colocalizing with nuclei highlighted by DAPI (blue).

© 2021 The Authors
